# Phase Angle of Bioelectrical Impedance Analysis as an Indicator for Diabetic Polyneuropathy in Type 2 Diabetes Mellitus

**DOI:** 10.1210/clinem/dgad737

**Published:** 2024-01-12

**Authors:** Lukas Schimpfle, Dimitrios Tsilingiris, Christoph M Mooshage, Zoltan Kender, Alba Sulaj, Ekatherina von Rauchhaupt, Julia Szendroedi, Stephan Herzig, Jens Goepfert, Jan Groener, Peter P Nawroth, Martin Bendszus, Sabine Heiland, Felix T Kurz, Johann M E Jende, Stefan Kopf

**Affiliations:** Department for Endocrinology, Diabetology, Metabolic diseases and Clinical Chemistry, University Hospital Heidelberg, 69120 Heidelberg, Germany; German Center for Diabetes Research (DZD), 85764 Munich-Neuherberg, Germany; Department for Endocrinology, Diabetology, Metabolic diseases and Clinical Chemistry, University Hospital Heidelberg, 69120 Heidelberg, Germany; German Center for Diabetes Research (DZD), 85764 Munich-Neuherberg, Germany; Department of Neuroradiology, University Hospital Heidelberg, 69120 Heidelberg, Germany; Department for Endocrinology, Diabetology, Metabolic diseases and Clinical Chemistry, University Hospital Heidelberg, 69120 Heidelberg, Germany; German Center for Diabetes Research (DZD), 85764 Munich-Neuherberg, Germany; Department for Endocrinology, Diabetology, Metabolic diseases and Clinical Chemistry, University Hospital Heidelberg, 69120 Heidelberg, Germany; German Center for Diabetes Research (DZD), 85764 Munich-Neuherberg, Germany; Department for Endocrinology, Diabetology, Metabolic diseases and Clinical Chemistry, University Hospital Heidelberg, 69120 Heidelberg, Germany; German Center for Diabetes Research (DZD), 85764 Munich-Neuherberg, Germany; Department for Endocrinology, Diabetology, Metabolic diseases and Clinical Chemistry, University Hospital Heidelberg, 69120 Heidelberg, Germany; German Center for Diabetes Research (DZD), 85764 Munich-Neuherberg, Germany; Institute for Diabetes and Cancer IDC and Joint Heidelberg-IDC Translational Diabetes Program, Helmholtz Center, 85764 Munich-Neuherberg, Germany; German Center for Diabetes Research (DZD), 85764 Munich-Neuherberg, Germany; Institute for Diabetes and Cancer IDC and Joint Heidelberg-IDC Translational Diabetes Program, Helmholtz Center, 85764 Munich-Neuherberg, Germany; NMI Natural and Medical Sciences Institute at the University of Tübingen, 72076 Tübingen, Germany; Zentrum für Diabetes und Hormonerkrankungen, 67433 Neustadt an der Weinstraße, Germany; Department for Endocrinology, Diabetology, Metabolic diseases and Clinical Chemistry, University Hospital Heidelberg, 69120 Heidelberg, Germany; German Center for Diabetes Research (DZD), 85764 Munich-Neuherberg, Germany; Department of Neuroradiology, University Hospital Heidelberg, 69120 Heidelberg, Germany; Department of Neuroradiology, University Hospital Heidelberg, 69120 Heidelberg, Germany; Department of Neuroradiology, University Hospital Heidelberg, 69120 Heidelberg, Germany; German Cancer Research Center, Radiology, 69120 Heidelberg, Germany; Department of Neuroradiology, University Hospital Heidelberg, 69120 Heidelberg, Germany; Department for Endocrinology, Diabetology, Metabolic diseases and Clinical Chemistry, University Hospital Heidelberg, 69120 Heidelberg, Germany; German Center for Diabetes Research (DZD), 85764 Munich-Neuherberg, Germany

**Keywords:** diabetic polyneuropathy, bioelectrical impedance analysis, magnetic resonance neurography, nerve conduction studies, phase angle, quantitative sensory testing

## Abstract

**Context:**

Due to the heterogenous clinical symptoms and deficits, the diagnosis of diabetic polyneuropathy (DPN) is still difficult in clinical routines, leading to increased morbidity and mortality.

**Objective:**

We studied the correlation of phase angle (PhA) of bioelectrical impedance analysis (BIA) with clinical, laboratory, and physical markers of DPN to evaluate PhA as a possible diagnostic method for DPN.

**Materials and methods:**

In this cross-sectional observational study as part of the Heidelberg Study on Diabetes and Complications, we examined 104 healthy individuals and 205 patients with type 2 diabetes mellitus (T2D), among which 63 had DPN. The PhA was calculated from multifrequency BIA. Nerve conduction studies, quantitative sensory testing (QST) and diffusion-weighted magnetic resonance neurography to determine fractional anisotropy (FA) reflecting peripheral nerve integrity were performed.

**Results:**

T2D patients with DPN had lower PhA values (5.71 ± 0.10) compared to T2D patients without DPN (6.07 ± 0.08, *P* = .007, + 6.1%) and healthy controls (6.18 ± 0.08, *P* < .001, + 7.9%). Confounder-adjusted analyses showed correlations of the PhA with conduction velocities and amplitudes of the peroneal (β=.28; β=.31, *P* < .001) and tibial nerves (β=.28; β=.32, *P* < .001), Z-scores of QST (thermal detection β=.30, *P* < .05) and the FA (β=.60, *P* < .001). Receiver-operating characteristic analysis showed similar performance of PhA in comparison to the mentioned diagnostic methods.

**Conclusion:**

The study shows that PhA is, in comparison to other test systems used, at least an equally good and much easier to handle investigator-independent marker for detection of DPN.

Sensorimotor distal diabetic polyneuropathy (DPN) is the most common complication affecting up to 50% of patients with diabetes mellitus (DM) (1). With treatment options still being limited (2), DPN leads to a significant increase in morbidity related to the development of diabetic foot syndrome and contributes to increased mortality following leg amputation (3).

Standard diagnostic methods for DPN include clinical scores such as the neuropathy disability score (NDS) and the neuropathy symptom score (NSS) (4, 5). However, clinical scores are of limited sensitivity and/or specificity, are investigator dependent, and fail to detect early manifestations of DPN (6). This results in underdiagnosis and often delayed detection when irreversible nerve damage already has occurred (7).

Methods of high specificity and sensitivity, which are restricted to specialized centers, include nerve conduction studies (NCS) and quantitative sensory testing (QST), which has been proven to be precise, reproducible, and suitable to detect subclinical neuropathic changes (8, 9). In addition, circulating biomarkers of DPN, such as the neurofilament light chain protein (NFL), predict the onset of DPN (10). The most sophisticated imaging method for the detection of structural nerve damage in DPN is magnetic resonance neurography (MRN) (11-13). Due to new imaging protocols with diffusion tensor imaging derived fractional anisotropy (FA), the accuracy of visualizing neural integrity has further increased (14). In patients with DPN, the FA was shown to be decreased in comparison to patients with DM without DPN and correlated well with clinical scores and electrophysiological measures (12, 15).

All methods and techniques mentioned earlier have certain disadvantages: NCS and QST are limited by patient's discomfort, time consumption, and availability (16). Additionally, NCS only allows detection of large fiber abnormalities in late stages of DPN, and QST is laborious and cost-intensive with a substantial impact of emotional disturbances (17). NFL and MRN are mostly unavailable in routine clinical care, and MRN is restricted to a number of individuals due to contraindications. Therefore, although a variety of highly sensitive and specific diagnostic tools exists for the diagnosis of DPN, a highly accessible diagnostic method that is easy to handle and does not cause discomfort of the person examined remains unavailable to date.

Bioelectrical impedance analysis (BIA) has been shown to be of high diagnostic value for assessment of disease severity in the context of malnutrition (18), liver cirrhosis (19, 20), cancer (21), chronic dialysis (22), or COVID-19 (23). It was also shown to be of predictive value for mortality in certain patient groups (19). BIA quantifies human body composition by applying alternating low-voltage electrical current to the lower and upper extremities (24). Phase angle (PhA) is the vector reflecting the angular phase shift of voltage and current (25). The PhA is considered to be a marker of cellular health with the theory of decreased voltage conduction due to disturbed membrane potentials (26). With regard to DM, there are several studies showing an association of the PhA with glycemic control (27) allowing differentiation of individuals with DM from glucose-tolerant controls (28-30). The application of BIA for the evaluation of DPN has not been evaluated yet.

Since neuronal fibers are very sensitive to membrane dysfunction (31), we argue that a test reflecting disturbed membrane potential in all tissues might be a useful tool, not only to support the notion of membrane potential disorders as cause of diabetic complications but also as a noninvasive, investigator-independent test for the presence of diabetic neuropathy (22). Therefore, a study was conducted to assess the possible value of this easy and noninvasive method when compared to several of the classical and well-established “gold standard” tests for DPN.

## Materials and Methods

### Study Design

This study is part of the Heidelberg Study on Diabetes and Complications (HEIST-DiC, local ethics number S-383/20161, identifier NCT03022721), approved by the local ethics committee and in accordance with the Declaration of Helsinki. After giving written informed consent, pseudonymized participants underwent clinical, laboratory, and electrophysiological examinations at the Department of Internal Medicine I. Patients with DM were diagnosed according to German national guidelines while glucose-tolerant controls received confirmatory oral glucose tolerance tests (32). MRN was conducted at the Department of Neuroradiology by experienced neuroradiologists who were blinded to clinical data.

### Participants

In this study we excluded participants of young age (under 18 years), active pregnancy, malignancy, spinal surgery, relevant disc protrusion, or chronic neurological diseases. Probands with risk profiles for neuropathy such as alcoholism, usage of neurotoxic agents, or relevant vitamin deficiencies and patients with severe cardiovascular, liver, and renal disease were excluded as well.

### Neuropathy Assessment

#### Clinical scores

For evaluation of DPN, the NSS, a standardized history taking for neuropathic symptoms, and the clinical assessment in terms of the NDS were used. The diagnosis of DPN was confirmed according to current guidelines of the German Society of Diabetology with either prominent signs (NDS > 5) or a combination of symptoms and signs (NDS > 2 and NSS > 3) (4, 33). As secondary criterion the *confirmed clinical DPN diagnosis* in accordance with the Toronto Consensus (34) was used, defined as the presence of neuropathic symptoms (NDS > 2) or signs (NSS > 2) as well as NCS below the 2.5 percentile determined from healthy controls.

#### QST

The protocol for QST included 13 different parameters that were compared to reference values issued by the German Research Network on Neuropathic Pain to create Z-scores adjusted to age and sex (9). For statistical analyses, the compound scores for mechanical and thermal detection and pain were calculated as described previously (11).

#### NCS

NCS (Viasys Healthcare VikingQuest; Viasys Healthcare GmbH, Höchberg, Germany) were performed on the right and left leg by a trained assistant under standardized conditions with skin temperature being at least 32 °C. The values obtained from motor tibial and peroneal nerves include nerve conduction velocities (NCVs), motor compound muscle action potentials (CMAPs), and distal motor latencies (DML). Sensory sural nerve measurements contain sensory nerve action potential amplitudes (SNAP) and NCV. For nondetectable sural NCV or SNAP due to severe DPN, we chose the lowest obtained value, respectively.

#### MRN, NFL

Participants eligible for magnetic resonance imaging received high-resolution MRN of the sciatic nerve at the level of distal right thigh using a 3 Tesla magnetic resonance imaging scanner (Magnetom TIM-TRIO, Siemens Healthineers, Erlangen, Germany). A high-resolution fat-saturated T2-weighted turbo spin echo 2D sequence and a diffusion tensor imaging sequence with an axial fat-suppressed, diffusion-weighted two-dimensional echo-planar sequence were applied as described previously (see Jende et al, 2021, 2020). The acquired images were pseudonymized, processed and analyzed automatically using the software Nordic BrainEx (Nordic Neurolab, Bergen, Norway) as described elsewhere (see Jende et al, 2021, 2020).

For quantification of serum NFL a Simoa immunoassay (Quanterix, Billerica, MA, USA) was used as described previously (10).

### Diabetic Nephropathy and Retinopathy Assessment

The diagnosis diabetic nephropathy confirmed according to the current Kidney Disease Improving Global Outcomes guideline with an increased albumin-creatinine ratio of over 30 mg/g in 2 morning spot urine samples (35). Diabetic retinopathy was diagnosed with single-field 45-degree fundoscopy by the same diabetologist with over 5 years of experience in the field. Images were taken on a nonmydriatic auto fundus camera (Nidek/Oculus AFC-230/210, NIDEK Co., Ltd., Japan) connected to a digital camera (21.8 megapixel full frame sensor, Canon EOS 5D Mark II, Canon Deutschland GmbH, Krefeld, Germany) as described before (36).

### BIA and PhA

The measurements were obtained using the BIACORPUS RX 4004 M (MEDI CAL HealthCare GmbH) multifrequency bioelectrical impedance device, and calculations were done with the integrated software according to Sergi’s equation (37). The examination was performed by trained assistants with over 5 years of experience following standard manufacturer's instructions.

All participants were in the fasted state and put in the supine position with 5 minutes of rest before measurements. Two electrodes were applied on each distal extremity, and measurements were obtained according to protocol. The PhA was automatically calculated with a standard formula: PhA = (reactance/resistance) × 180°/π (22).

### Statistical Analysis

Statistical data analyses were performed using IBM SPSS Statistics 27 (SPSS Inc., Chicago, IL, USA). If not stated differently, values are given in means with standard error. For comparison of categorical variables, Chi-squared test was used. Estimated marginal means were calculated with linear models after adjustment for confounding variables. For group comparisons of data with Gaussian distribution (Shapiro-Wilk test), t-tests or ANOVA with Bonferroni correction were used, while for data without Gaussian distribution, Mann-Whitney or Kruskal-Wallis tests were used. For correlation analysis we used Pearson or Spearman correlation coefficients depending on normal distribution. Multivariable linear regression models were performed giving the unstandardized (B) and standardized (β) partial correlation coefficients. The level of significance for all tests was defined at a 2-tailed *P* < .05.

## Results

### Demographic Results

In our study we included 104 healthy control participants (age: 54.3 ± 1.21; 71 females and 32 males) and 205 patients with type 2 DM (T2D) (age: 61.8 ± 0.98; 75 females and 130 males) among which 63 patients had clinical DPN (age: 67.3 ± 0.97; 17 females and 46 males) while 142 presented with T2D without DPN (58 females and 84 males). Anthropometric measures of participants are given in Table 1. Considering diabetes medication, there was no difference among patients with and without DPN (Supplementary Table S1) (38). PhA values did not differ when participants were grouped according to diagnosis of nephropathy and retinopathy (Supplementary Table S2) (38). The PhA was 7.9% lower in the group with DPN in comparison to controls and 6.1% lower in comparison to patients with T2D without DPN. Due to heterogenous groups and possible confounding factors (see Table 1), we adjusted the PhA comparisons for age, sex, body mass index (BMI), duration of diabetes, hemoglobin A1c (HbA1c), glomerular filtration rate (GFR), and triglycerides resulting in similar values (6.23 ± 0.09 vs 6.04 ± 0.06 vs 5.74 ± 0.10, *P* = .003).

**Table 1.** Characteristics of different groups

	Controls (n = 104)	T2D without DPN (n = 142)	T2D with DPN (n = 63)	*P*-values (ANOVA)
Age (years)	54.3 ± 1.2*^a,b^*	61.8 ± 1.0*^a,c^*	67.3 ± 1.0*^b,c^*	<.001
Sex (female/male)	71/32*^a,b^*	58/84*^a^*	17/46*^b^*	<.001
Duration of diabetes	0*^a,b^*	9.2 ± .7*^a,c^*	12.3 ± 1.3*^b,c^*	<.001
BMI (kg/m²)	27.8 ± 0.6*^a,b^*	31.1 ± 0.5*^a^*	31.3 ± 0.7*^b^*	<.001
HbA1c %	5.4 ± 0.1*^a,b^*	7.2 ± 0.1*^a^*	7.3 ± 0.2*^b^*	<.001
HbA1c (mmol/mol)	35.6 ± 0.4*^a,b^*	54.4 ± 1.3*^a^*	56.0 ± 1.6*^b^*	<0.001
GFR (mL/min/1.73m²)	95.7 ± 1.2*^a,b^*	87.7 ± 1.7*^a^*	85.6 ± 2.1*^b^*	<.001
Triglycerides (mg/dL)	105.8 ± 5.0*^a,b^*	195.5 ± 17.4*^a^*	180.4 ± 15.9*^b^*	<.001
OAD (%)	0	87 (61)	40 (63)	<.001*^d^*
Insulin therapy (%)	0	41 (29)	24(38)	<.001*^d^*
Phase angle (degrees)	6.20 ± 0.08*^b^*	6.07 ± 0.08*^c^*	5.72 ± 0.10*^b,c^*	.002
Adj. phase angle (degrees)*^e^*	6.22 ± 0.09*^b^*	6.04 ± 0.06*^c^*	5.74 ± 0.10*^b,c^*	.003

Abbreviations: BMI, body mass index; DPN, diabetic polyneuropathy; GFR, glomerular filtration rate; HbA1c, hemoglobin A1c; OAD, oral antidiabetic drug; T2D, type 2 diabetes mellitus.

Data shows anthropometric properties and phase angle values of the control group and groups with type 2 diabetes mellitus with and without diabetic polyneuropathy according German national guidelines. Values are given in means ± standard error or percentage with *P*-values of ANOVA on the right. Footnotes show if significance level of *P* < .05 is reached in post hoc Bonferroni correction, level of significance *P* < .05.

^*a*^Difference (*P* < .05) in post hoc analysis (Bonferroni correction) between controls and T2D without DPN.

^*b*^Difference (*P* < .05) in post hoc analysis (Bonferroni correction) between controls and T2D with DPN.

^*c*^Difference (*P* < .05) in post hoc analysis (Bonferroni correction) between T2D with and without DPN.

^*d*^Chi-square.

^*e*^Phase angle adjusted for age, sex, BMI, duration of diabetes, HbA1c, GFR, and triglycerides (estimated marginal means).

### Neuropathic Analysis

#### Quantitative sensory testing

Clinically, QST compound Z-scores showed reduced thermal detection (−0.54 ± 0.09 vs −0.70 ± 0.07 vs −1.51 ± 0.11), mechanical detection (−0.19 ± 0.11 vs −0.59 ± 0.10 vs −2.42 ± 0.25), and reduced mechanical pain sensation (1.04 ± 0.09 vs 0.77 ± 0.08 vs 0.02 ± 0.18) in patients with DPN in comparison to both other groups (Supplementary Table S3 and 4) (38). Thermal pain sensation was not significantly different.

**Figure 1.**
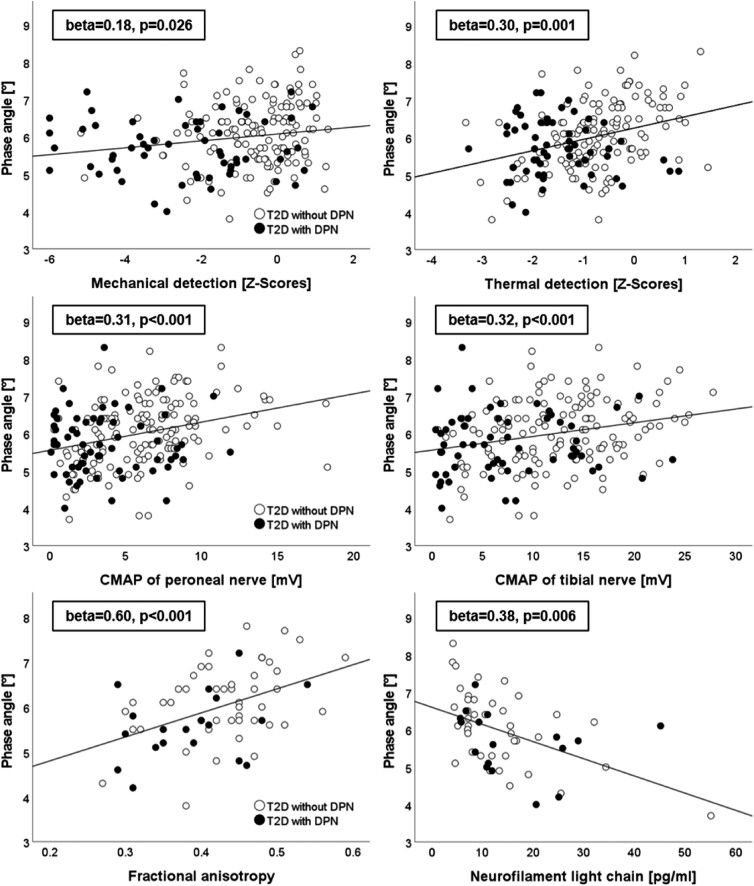
Association of phase angle with nerve conduction studies (middle), compound Z-scores of quantitative sensory testing (top), fractional anisotropy (bottom left), and neurofilament light chain (bottom right). Statistical values are given in standardized β with *P*-values from multivariate regression models. Abbreviations: CMAP, compound muscle action potential.

In correlation analysis, the PhA was positively correlated with compound Z-scores for thermal (r = 0.33, *P* < .001) and mechanical (r = 0.20, *P* = .005) detection (Fig. 1, top) while thermal and mechanical pain showed no association (Table 2). Multivariate analysis with age, sex, BMI, duration of diabetes, HbA1c, GFR, and triglycerides as independent variables showed similar results for mechanical [B = 0.35 95% confidence interval (CI) 0.04-0.66, β=.18, *P* = .026] and thermal detection scores (B = 0.32 95% CI 0.14-0.51, β=.30, *P* = .001).

**Table 2.** Associations of the phase angle with neuropathic parameters (only T2D, n = 205)

	Correlation analysis		Regression analysis^1^		
	R	*P*-value	B (95% CI)	β	*P*-value
Peroneal NCV (m/s)	0.11^p^	.110	**1.93** (0.93-2.93)	.**28**	**<**.**001**
Peroneal CMAP (mV)	**0**.**32^s^**	**<**.**001**	**1.25** (0.63-1.87)	.**31**	**<**.**001**
Peroneal DML (ms)	**−0**.**16^s^**	.**025**	−0.67 (−0.14-0.06)	−.15	.070
Tibial NCV (m/s)	0.10^p^	.153	**1.66** (0.76-2.56)	.**28**	**<**.**001**
Tibial CMAP (mV)	**0**.**26^s^**	**<**.**001**	**2.31** (1.18-3.44)	.**32**	**<**.**001**
Tibial DML (ms)	**−0**.**14^s^**	.**043**	**−0.73** (−1.33 to −.13)	**−**.**21**	.**017**
Sural NCV (m/s)	**0**.**22^s^**	.**002**	**4.29** (1.90-6.69)	.**28**	.**001**
Sural SNAP (µV)	**0**.**19^s^**	.**005**	0.51 (−0.07-1.09)	.13	.084
Z-score thermal detection (SD)	**0**.**33^p^**	**<**.**001**	**0.32** (.14-0.51)	.**30**	.**001**
Z-score thermal pain (SD)	0.09^s^	.221	0.18 (−0.01-0.38)	.16	.069
Z-score mechanical detection (SD)	**0**.**20^s^**	.**005**	**0.35** (0.04-0.66)	.**18**	.**026**
Z-score mechanical pain (SD)	0.13^p^	.064	0.17 (−0.06-0.40)	.13	.149
FA (n = 65)	**0**.**45^p^**	**<**.**001**	**0.05** (0.03-0.08)	.**60**	**<**.**001**
NFL (n = 57)	**−0**.**48^p^**	**<**.**001**	**−4.01** (−6.84 to −1.19)	**−**.**38**	.**006**

	Binary logistic regression*^a^*		B	Expo (B) (95% CI)	*P*-value
DPN according to NDS/NSS*^b^*			−0.59	.56 (.34-.91)	.**019**
Confirmed clinical DPN*^c^*			−0.86	.42 (.26-.68)	**<**.**001**

Abbreviations: CI, confidence interval; CMAP, compound muscle action potential; DML, distal motor latency; DPN, diabetic polyneuropathy; FA, fractional anisotropy, NCV, nerve conduction velocity; NDS, neuropathy disability score; NFL, neurofilament light chain; NSS, neuropathy symptom score; SNAP, sensory nerve action potential; T2D, type 2 diabetes mellitus.

Associations of the phase angle with neuropathic measurements in individuals with T2D (n = 205). Univariate correlation analysis is shown on the left with r-values of Pearson or Spearman analysis, significance level *P* < .05. Data on the right shows results of multivariate regression models with dependent variables given in the leftmost column and independent variables including phase angle, age, sex, body mass index, duration of diabetes, hemoglobin A1c, glomerular filtration rate, and triglycerides. Results of the phase angle are reported as unstandardized B-values with confidence intervals and standardized β coefficient with significance level of *P* < .05. The bottom shows binary logistic regression models with the same independent variables and DPN according to NDS/NSS and according to NDS/NSS plus NCS. All significant results were written in bold.

^*a*^Independent variables: age, sex, body mass index, duration of diabetes, hemoglobin A1c, glomerular filtration rate, and triglycerides.

^*b*^Yes = 1, No = 0, Criteria: NDS > 5 or NSS > 3 and NDS > 2.

^*c*^Yes = 1, No = 0, Criteria: NDS > 2 or signs NSS > 2 plus NCS < 2.5 percentile.

^p^Pearson.

^s^Spearman.

#### NCS

NCS showed decreased parameters of motor fibers such as peroneal and tibial NCV/CMAP and decreased parameters of sensory fibers such as sural NCV and SNAP in patients with DPN in comparison to controls and patients with T2D without DPN [Supplementary Table S3 (2, 3) and 4 (38)], while DML showed no significant differences.

Univariate analysis showed correlations of the PhA with motor parameters such as peroneal CMAP (r = 0.32, *P* < .001), peroneal DML (r = −0.16, *P* = .025), tibial CMAP (r = 0.26, *P* < .001), and tibial DML (r = −0.14, *P* = .043) and with sensory NCV (r = 0.22, *P* = .002) and SNAP (r = 0.19, *P* = .005) of the sural nerve. Multivariate regression models with the mentioned confounding factors revealed furthermore associations of the PhA with NCV of the peroneal (B = 1.93, 95% CI 0.93-2.93, *P* < .001) and tibial nerve (B = 1.66, 95% CI 0.76-2.56, *P* < .001, see Table 2).

Binary logistic regression also showed an association of the PhA with the diagnosis of DPN according to German national guidelines [Expo(B) = 0.56, 95% CI 0.34-0.91, *P* = .019] and according to Toronto criteria [Expo(B) = 0.42, 95% CI 0.26-0.68, *P* < .001].

#### MRN, NFL

MRN was performed on 93 individuals including 28 controls (21 females and 7 males), 45 T2D patients without DPN (18 females and 27 males), and 20 patients with DPN (3 females and 17 males). The integrity of nerve fibers measured as FA was shown to be highest in the control group (0.50 ± 0.01) with lower values in the group with T2D without DPN (0.43 ± 0.01) and lowest in the group with DPN (0.39 ± 0.02).

PhA values were positively correlated with the FA (r = 0.45, *P* < .001) in univariate analysis (Fig. 1, bottom left). This association remained significant in a linear regression model with age, sex, BMI, duration of diabetes, HbA1c, GFR, and triglycerides as independent variables (B = 0.05, 95% CI 0.03-0.08, β=.60, *P* < .001).

Lastly, the PhA showed a negative correlation with the serological marker of axonal damage NFL (r = −0.48, *P* < .001) that remained consistent in multivariate calculations (B = −4.01, 95% CI −6.84 to −1.19, β= −.38, *P* = .006).

#### ROC analysis

In order to test the potential of the PhA to discriminate between patients with and without DPN, ROC analysis was performed with PhA and QST Z-scores. The state variable the Toronto consensus criterion *confirmed clinical DPN* was used to assess both electrophysiological and clinical diagnosis of DPN.

**Figure 2.**
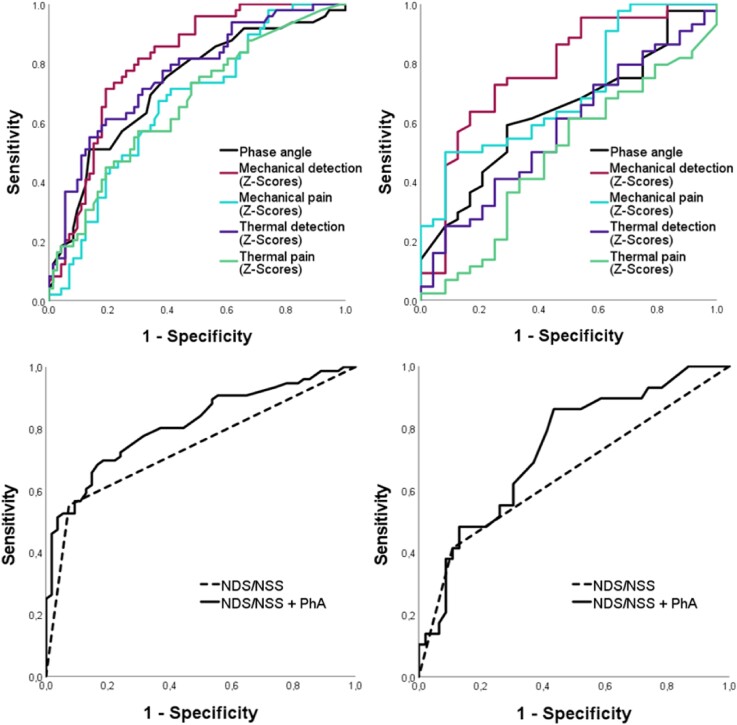
ROC curves in males (left) and females (right) with T2D. A state variable, confirmed clinical DPN according to the Toronto Consensus Criteria, was used. Top images: Phase angle in black with compound Z-scores of QST namely mechanical detection Z-scores in dark red, mechanical pain Z-scores in turquoise, thermal detection Z-scores in purple, and thermal pain Z-scores in green. Bottom images: Clinical scores (NDS > 5 or NSS > 3 and NDS > 2) in black dotted line in comparison to a combination of phase angle and mentioned clinical scores criteria in solid black. Abbreviations: AUC, area under the curve; CI, confidence interval; DPN, diabetic polyneuropathy; NDS, neuropathy disability score; NSS, neuropathy symptom score; PhA, phase angle; QST, quantitative sensory testing; ROC, receiver-operating characteristic; T2D, type 2 diabetes mellitus.

For males the PhA with a cut-off value of 6.15° showed a sensitivity of 76.5% and a specificity of 60.3% with an area under the curve (AUC) of 0.726 (95% CI 0.633-0.819, *P* < .001; see Fig. 2, top left). A value of 6.75° had a sensitivity of 51.0% and a specificity of 86.3%. For females, the cut-off value of 5.45° revealed a sensitivity of 61.4% and a specificity of 62.5% and an AUC of 0.637 (95% CI 0.503-0.772, *P* = .045; see Fig. 2, top right).

**Table:** The ROC analysis determines the predictive value of each test procedure (mechanical pain, thermal pain, phase angle, NDS/NSS, and thermal detection) for the presence of diabetic polyneuropathy (DPN) compared to the standardized Toronto criteria, using the calculated AUC (shown in Figure 2). Significant p-values are highlighted in bold.

	AUC	95% CI	Significance
Males (n = 130)			
Thermal pain	0.664	0.566-0.762	**0.001**
Mechanical pain	0.666	0.569-0.762	**0.001**
Phase angle	0.726	0.633-0.819	**<0.001**
NDS/NSS	0.739	0.654-0.825	**<0.001**
Thermal detection	0.762	0.676-0.849	**<0.001**
Mechanical detection	0.805	0.728-0.882	**<0.001**
NDS/NSS + PhA	0.814	0.742-0.886	**<0.001**
Females (n = 75)			
Thermal pain	0.489	0.342-0.636	0.885
Thermal detection	0.578	0.436-0.719	0.282
Phase angle	0.637	0.503-0.772	**0.045**
NDS/NSS	0.653	0.519-0.786	**0.027**
Mechanical pain	0.705	0.576-0.833	**0.002**
NDS/NSS + PhA	0.740	0.625-0.854	**0.001**
Mechanical detection	0.777	0.655-0.900	**<0.001**

When combining PhA with German national diagnostic criteria of DPN, the area under the curve increases further in comparison to PhA alone (AUC in males from 0.726 to 0.814 and females from 0.637 to 0.740; Fig. 2, top) and clinical scores alone (AUC in males from 0.739 to 0.814 and females from 0.653 to 0.740; Fig. 2, bottom).

For assessment of DPN severity, we performed a subgroup analysis of neuropathic parameters within patients with DPN. In multivariate regression analyses, only NCV of the tibial nerve and tibial DML showed significant results (Supplementary Table S5) (38). Sex-specific subgroup analyses in patients with T2D showed more relevant associations of neuropathic measurements with PhA values in males than in females (Supplementary Tables S6 and S7) (38).

## Discussion

### Main Findings

To our knowledge, this study was the first to apply the PhA in the context of DPN. We found that PhA is lower in individuals with DPN compared to healthy controls or patients with T2D without DPN. There was no difference for PhA values between controls and T2D patients without DPN, thus parameters other than glycemia related to the onset of DPN are reflected by a decrease in PhA. Further analyses in patients with T2D were performed using a wide spectrum of clinical, electrophysiological, serological, and radiological examinations of DPN reflecting neuronal integrity and axonal damage. Here, we found PhA values to be associated with clinical scores (NDS/NSS), NCS, QST Z-scores, FA, and NFL independent of confounding parameters such as age, sex, BMI, duration of diabetes, glycemic status, renal function, and lipid levels. For the diagnosis *confirmed clinical DPN,* ROC analysis in male patients showed similar sensitivity and specificity values than thermal QST Z-scores, the parameter of highest sensitivity for detecting early DPN (8). This is the first study showing associations of the PhA with established neuropathy markers in DPN. Being investigator independent, cost-effective, noninvasive, practical, and without patient discomfort, it could be implemented in future DPN diagnosis.

### Potential Role of PhA in Common Diagnostic Methods of DPN

Our results indicate that PhA is associated with multiple determinants of DPN. The ROC analysis showed comparable sensitivity to QST Z-scores, making it a possible screening instrument in the clinical setting of DPN. The highest correlation exists with thermal detection compound Z-scores reflecting C-fiber dysfunction, a pathology typically seen in early DPN supporting the early screening value of PhA (8, 31). Nevertheless, PhA values alone were shown to be inferior to mechanical detection Z-scores. When adding PhA to clinical DPN diagnosis with NDS/NSS, similar results as with mechanical detection scores were achieved (see Fig. 2, bottom). Hence, for best sensitivity, a combination of PhA with clinical scores would be useful.

To assess whether PhA may also serve as a marker of disease severity, we performed subgroup analysis within the group of DPN where tibial NCV and DML remained significant after confounder analysis (Supplementary Table S5) (38). PhA values could therefore also reflect motor involvement with beginning muscular atrophy; however, assessment of severity and changes in DPN is limited (34) and longitudinal analysis is needed.

### Overview of Available Literature of PhA in T2D

There are limited comparable studies regarding the PhA in patients with T2D; none were designed to examine diabetic complications. In comparison to healthy populations, lower PhA values in general were found in patients with T2D (27-30). In this study, we also detected decreased PhA values in patients with T2D, however, only in the subgroup with DPN after controlling for confounding factors including glycemic status. Hence, we assume that previous associations made without differentiating for diabetic complications were most likely the result of different characteristics of patients with DPN in the cohorts.

### Relationship of PhA With Characteristics of DPN

#### PhA as determinant of risk factor for DPN

Since PhA is considered to decrease with increasing age, BMI, and decreased states of health (22), a plausible explanation for the observed associations would implicate the PhA as an indicator of risk factors for developing DPN (39). However, in our regression models we eliminated most common confounding risk factors for DPN; therefore, PhA values should reflect other characteristics of DPN. On a pathophysiological level, PhA has been linked to inflammation (40), oxidative stress, and cell damage (26), keystones in the development of DPN (31).

Another major risk factor for DPN is male sex. In a sex-specific analysis of our cohort, correlations of neuropathic measures with PhA were stronger in males than in female individuals (Supplementary Tables S6 and S7) (38). While male individuals are at higher risk for developing DPN, females express earlier symptoms and are more likely to suffer from painful DPN (41). While little is known about sex-specific changes of DPN, our results might differentiate sex-specific diagnostic aspects. Accordingly, correlations of the PhA dominating in males may reflect different pathophysiological mechanisms in DPN development between both sexes (42); however, our study was not designed for sex-specific analysis.

#### Associations of PhA on a pathophysiological level

The associations of PhA values with markers of neuronal integrity, such as FA obtained from MRN and the serological parameter NFL, indicates that changes on a neural level are also reflected by the method. The FA is based on the principle that water diffusion in nerves is restricted by myelin and cell membranes (15). The hypothesis that PhA may reflect cellular integrity has been stated before and is well established with muscle mass and sarcopenia (20, 22, 25, 26). However, neither clinical nor preclinical studies exist examining the PhA in the nervous system in particular due to BIA being an indirect and global assessment of body composition.

Considering the different nerve types involved in DPN, our data suggests that PhA measurement is rather reflecting A-beta- and C-fiber damage (QST compound detection scores) than A-delta fibers (QST compound pain scores) (9, 11). The significance of these findings is unclear; nevertheless, it can be hypothesized that the missing correlation is due to the subjective nature of pain, and more objective small-fiber studies are needed for the future. PhA associations in patients with DPN could reflect muscle atrophy in progressed motor polyneuropathy (43, 44). Our data supports this hypothesis, since correlations of the PhA with motor NCVs and CMAPs were more prominent in comparison with sensory NCS parameters (16). However, muscular atrophy was not specifically assessed in our study, which would be necessary to investigate this hypothesis. Nevertheless, independent associations with NFL, a marker for axonal impairment in neuropathies such as DPN (10, 45), highlight a connection of PhA measurements with neuronal integrity in DPN.

### Limitations

One main limitation of our study is the cross-sectional design; therefore, a follow-up analysis is planned for longitudinal analyses. Furthermore, our groups were different for age and sex so we performed age-adjusted and sex-specific analyses to increase meaningfulness of the presented data. Due to the observational study design, our group characteristics reflect the general distribution of patients with DPN (elderly males). Multiple confounders were included in statistical regression models, revealing independent correlations.

Despite using broad DPN diagnostic methods, confocal microscopy and intraepidermal nerve fiber density are missing when assessing small fiber associations and are to be implemented in future studies. Additionally, we did not test for vitamin B12 levels, although the exclusion of individuals with anemia or high mean erythrocyte corpuscular volume renders it unlikely that this has confounded our results. Lastly, general limitations of BIA include missing standardization and reference values (46). Despite that most factors influencing PhA were included in our statistical analysis, hydration status could not be assessed in every patient. Still, risk groups for edema such as patients with severe renal, liver, and cardiac disease were excluded, and all participants were fasted.

## Conclusion

In conclusion, this study is the first to find decreased BIA-derived PhA values in T2D patients with DPN compared to those with T2D without DPN and healthy controls. We found independent associations of PhA with clinical, electrophysiological, radiological, and serological determinants of DPN. Although longitudinal studies are needed to further strengthen this notion, our data suggests that PhA may be useful for DPN screening or for identifying patient subgroups at risk for DPN to prevent further complications.

## Data Availability

Some or all datasets generated during and/or analyzed during the current study are not publicly available but are available from the corresponding author on reasonable request.

## Clinical Trial Information

ClinicalTrials.gov Registration no. NCT03022721.

## Abbreviations

AUC: area under the curve
BIA: bioelectrical impedance analysis
BMI: body mass index
CMAP: compound muscle action potentials
DM: diabetes mellitus
DML: distal motor latencies
DPN: diabetic polyneuropathy
FA: fractional anisotropy
GFR: glomerular filtration rate
HbA1c: hemoglobin A1C
MRN: magnetic resonance neurography
NCS: nerve conduction studies
NCV: nerve conduction velocity
NDS: neuropathy disability score
NFL: neurofilament light chain
NSS: neuropathy symptom score
PhA: phase angle
QST: quantitative sensory testing
ROC: receiver-operating characteristic
T2D: type 2 diabetes mellitus
